# *CsXDH1* gene promotes caffeine catabolism induced by continuous strong light in tea plant

**DOI:** 10.1093/hr/uhad090

**Published:** 2023-05-04

**Authors:** Qianhui Tang, Keyi Liu, Chuan Yue, Liyong Luo, Liang Zeng, Zhijun Wu

**Affiliations:** College of Food Science, Southwest University, Chongqing 400715, China; Chongqing Key Laboratory of Speciality Food Co-Built by Sichuan and Chongqing, Southwest University, Chongqing 400715, China; Integrative Science Center of Germplasm Creation, Southwest University, Chongqing 401329, China; Tea Research Institute, Southwest University, Chongqing 400715, China; College of Food Science, Southwest University, Chongqing 400715, China; Chongqing Key Laboratory of Speciality Food Co-Built by Sichuan and Chongqing, Southwest University, Chongqing 400715, China; Integrative Science Center of Germplasm Creation, Southwest University, Chongqing 401329, China; Tea Research Institute, Southwest University, Chongqing 400715, China; College of Food Science, Southwest University, Chongqing 400715, China; Chongqing Key Laboratory of Speciality Food Co-Built by Sichuan and Chongqing, Southwest University, Chongqing 400715, China; Integrative Science Center of Germplasm Creation, Southwest University, Chongqing 401329, China; Tea Research Institute, Southwest University, Chongqing 400715, China; College of Food Science, Southwest University, Chongqing 400715, China; Chongqing Key Laboratory of Speciality Food Co-Built by Sichuan and Chongqing, Southwest University, Chongqing 400715, China; Integrative Science Center of Germplasm Creation, Southwest University, Chongqing 401329, China; Tea Research Institute, Southwest University, Chongqing 400715, China; College of Food Science, Southwest University, Chongqing 400715, China; Chongqing Key Laboratory of Speciality Food Co-Built by Sichuan and Chongqing, Southwest University, Chongqing 400715, China; Integrative Science Center of Germplasm Creation, Southwest University, Chongqing 401329, China; Tea Research Institute, Southwest University, Chongqing 400715, China; College of Food Science, Southwest University, Chongqing 400715, China; Chongqing Key Laboratory of Speciality Food Co-Built by Sichuan and Chongqing, Southwest University, Chongqing 400715, China; Integrative Science Center of Germplasm Creation, Southwest University, Chongqing 401329, China; Tea Research Institute, Southwest University, Chongqing 400715, China

## Abstract

Tea plant (*Camellia sinensis*) is an important cash crop with extensive adaptability in the world. However, complex environmental factors force a large variation of tea quality-related components. Caffeine is essential for the formation of bitter and fresh flavors in tea, and is the main compound of tea that improves human alertness. Continuous strong light stimulation was observed to cause caffeine reduction in tea leaves, but the mechanism is not clear. In this study, the response of tea plant to light intensity was analysed mainly by multi-omics association, antisense oligodeoxynucleotide (asODN) silencing technique, and *in vitro* enzyme activity assay. The results revealed multiple strategies for light intensity adaptation in tea plant, among which the regulation of chloroplasts, photosynthesis, porphyrin metabolism, and resistance to oxidative stress were prominent. Caffeine catabolism was enhanced in continuous strong light, which may be a light-adapted strategy due to strict regulation by xanthine dehydrogenase (XDH). asODN silencing and enzymatic activity assays confirmed that CsXDH1 is a protein induced by light intensity to catalyze the substrate xanthine. *CsXDH1* asODN silencing resulted in significant up-regulation of both caffeine and theobromine in *in vitro* enzyme activity assay, but not *in vivo*. CsXDH1 may act as a coordinator in light intensity adaptation, thus disrupting this balance of caffeine catabolism.

## Introduction

Caffeine (1,3,7-trimethylxanthine), a xanthine alkaloid compound, is particularly abundant in a few plants, most typically corps used to process beverages, such as tea plant (*Camellia sinensis*), coffee (*Coffea arabica*) and cocoa (*Theobroma cacao*) [1]. Caffeine is an important contributor to the bitterness of tea, and also gives tea a refreshing taste by complexing with catechins and theaflavins [2, 3]. Moderate caffeine intake can improve alertness, attention, and memory as well as reduce fatigue [4, 5]. In contrast, excessive intake of caffeine will cause insomnia, palpitations, and other uncomfortable symptoms [6]. Especially for pregnant women and children, excessive caffeine intake will cause rapid heartbeat and elevated blood pressure, and even dizziness and nausea in severe cases [6, 7]. At present, many aspects of caffeine biosynthesis and catabolism mechanisms in tea plant remain to be elucidated [1, 8]. If the caffeine content in raw materials can be effectively controlled, it will help broaden the market demand for tea.

In tea plant, more than 99% of caffeine exists in leaf tissue, the content is about 2 ~ 5% of leaf dry weight [9–11]. For the biosynthesis of caffeine, both purine ring and methyl donor are required. Purine ring of caffeine is formed by *de novo* pathway and salvage pathway of purine [12, 13]. The *de novo* pathway of purines is initiated by ribose-5-phosphate, which is catalyzed by 11-steps enzymatic process to form inosine monophosphate (IMP) [12]. The salvage pathway that supplies the caffeine purine ring is the reuse process of free bases or nucleosides [12, 13]. It is suggested that adenine is considered to be the most efficient precursor for caffeine biosynthesis [9]. The supply of purine rings further forms xanthosine, which is the starting substrate for the core pathway of caffeine biosynthesis [12]. Under the catalysis of *N*-methyltransferases (NMTs) and *N*-methylnucleotidase (N-MeNase), xanthosine successively forms 7-methylxanthosine, 7-methylxanthine, theobromine (3,7-dimethylxanthine) or paraxanthine (1,7-dimethylxanthine), and caffeine (1,3,7-trimethyl xanthine) [1, 12]. Caffeine synthesis via the precursor theobromine is considered to be the main route [12]. The conversion of theobromine to caffeine is catalyzed by caffeine synthase, which has been highly purified and recombinantly tested [14, 15]. Theobromine has similar functions to caffeine and is about 10% of caffeine content in tea plant [16]. *S*-adenosyl-L-methionine of SAM cycle acts as a methyl donor in caffeine biosynthesis [17, 18].

The weak catabolism of caffeine in the young tea leaves is presumed to be the main reason for its accumulation in this tissue [19]. The catabolism of caffeine is more pronounced in mature and aged leaves than in young leaves, and the conversion of caffeine to theophylline may be the main rate-limiting step of catabolism [19]. According to some tracer experiments [20, 21], the main pathways of caffeine catabolism was proposed to be from caffeine in order to produce theophylline and 3-methylxanthine or 1-methylxanthine, and then degraded by xanthine catabolism, finally producing glyoxylate and CO_2_ + NH_3_. The catalytic enzymes of caffeine catabolism of tea plant mainly include *N*-demethylases (NDMs), xanthine dehydrogenase (XDH), and enzymes related to urate degradation [21]. Theophylline feeding produces theobromine and caffeine, suggesting a possible bidirectional regulation of the purine ring in caffeine catabolism [19, 22]. In addition, purine catabolism in tea plant is also executed through the direct conversion of hypoxanthine, xanthosine, and guanine to xanthine [23].

Tea plant was first discovered and started to be cultivated in China, and nowadays it has been introduced to dozens of countries around the world as an important cash crop. The geographical areas of tea planting involve plains, hills, mountains, and plateaus in the temperate and tropical regions, which shows the excellent environmental suitability of tea plants [24, 25]. However, the complex environmental factors force the differentiation of tea components, thus affecting the sensory quality characteristics and health benefits. Drought will cause a decrease in total polyphenols, aqueous extracts, and free amino acids of tea leaves [26, 27]. Sub-high temperature causes the decrease of theanine in tea leaves, while low temperature affects the biosynthesis of chlorophylls and carotenoids [28, 29]. The accumulation of flavonoids, theanine, chlorophyll, and caffeine in tea plant is correlated with light intensity. Strong light helps the formation of flavonoids, while shade is better for the accumulation of theanine, chlorophyll, and caffeine [8, 30–33]. Currently, with the purification of key enzymes and the development of multi-omics, the regulatory pathways of structural genes in caffeine metabolism of tea plant have been gradually clarified [14, 22, 34, 35]. However, the molecular basis for the association of caffeine accumulation with light intensity is still unclear.

In this study, the co-analysis of metabolome, transcriptome, and proteome was used to evaluate the response of tea plant to light intensity. In addition, the validation analysis of the regulation mechanism of light-induced caffeine catabolism in tea plant was mainly based on the antisense oligodeoxynucleotide (asODN) silencing, *in vitro* enzyme activity, and subcellular localization assays.

**Figure 1:**
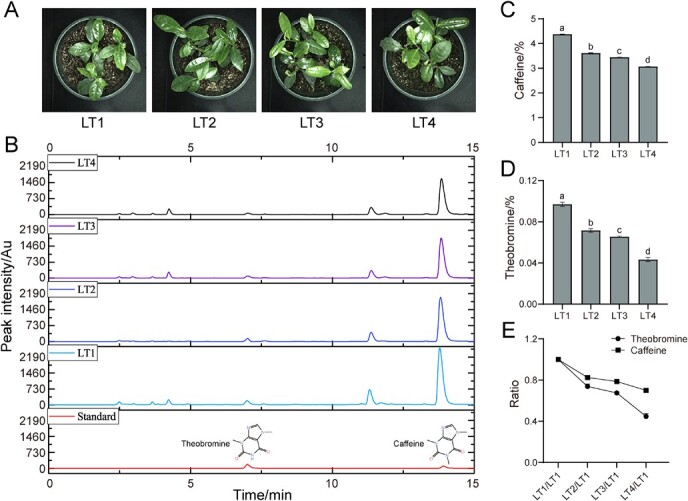
Tea plant treated with gradient light intensity. **A** Phenotype of tea plants after treatment. **B** Liquid chromatogram of caffeine and theobromine contents in treated samples. **C** Caffeine content in treated samples. **D** Theobromine content in treated samples. **E** Comparison of caffeine and theobromine content in treated samples. The light intensities of LT1, LT2, LT3, and LT4 were 15 μmol·m^−2^·s^−1^, 160 μmol·m^−2^·s^−1^, 200 μmol·m^−2^·s^−1^, and 240 μmol·m^−2^·s^−1^, respectively.

## Results

### Strong light induces the decrease of caffeine and theobromine content in tea plant

Previous studies have found that light intensity is an important environmental factor affecting the accumulation of caffeine in tea leaves [30–32]. In this study, four groups of light conditions (LT1, LT2, LT3, and LT4) were set up to test the correlation between changes in light intensity gradients and the phenotype and metabolite formation in tea plant. Tea plant phenotypes after a period of treatment (14 d): tea seedlings grew weakly under weak light intensity (LT1), while other tea seedlings grew well under stronger light intensity (LT2, LT3, and LT4); with increasing light intensity (from LT1 to LT4), the leaf color of tea seedlings tended to change from dark green to bright green and the chlorophyll content in leaves was gradually decreasing (Fig. 1A; Table S1, see online supplementary material). Subsequently, the contents of caffeine and theobromine as a percentage of the dry weight of tea leaves were detected by high performance liquid chromatography (HPLC). Light intensity showed a significant negative linear correlation with caffeine or theobromine content in tea leaves. The contents of caffeine and theobromine gradually decreased with increasing light intensity (Fig. 1B, C, and D). Both caffeine and theobromine contents had the greatest variability between LT1 and LT4 treated samples. Compared with LT1 treatment, the content of caffeine and theobromine in LT4 treated samples decreased by 29.96% and 55.24%, respectively (Fig. 1E). Compared to caffeine, the decreasing trend of theobromine content was more obvious with increasing light intensity.

### Integrating transcriptome and proteome sequencing to analyse the response of tea plant to strong light signal

**Figure 2:**
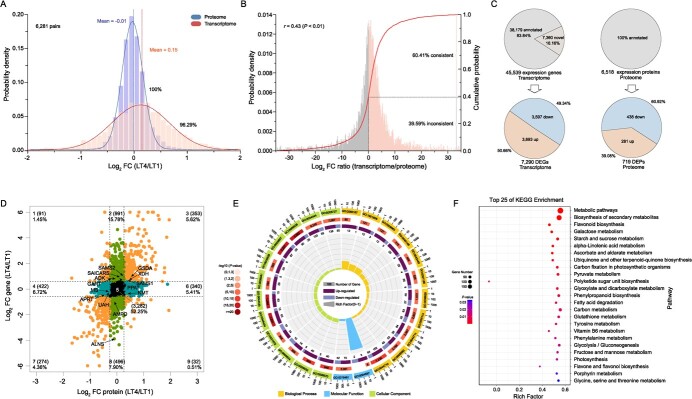
Integration analysis of transcriptome and proteome sequencing in light-treated tea plants. **A** Distribution density of expression data ratio (converted by log_2_) between treatment group (LT4) and control group (LT1). **B** Comparison of gene transcription and protein expression consistency. Data used log_2_ (LT4/LT1). A positive ratio between two omics was considered be consistent expression. **C** Screening of differentially expressed genes (DEGs) and differentially expressed proteins (DEPs). **D** Nine-quadrant distribution of expressed gene and protein pairs (EGPPs) expression data. LT4/LT1 fold thresholds were 1.5 and 1.2, respectively. **E** GO annotation of expression-consistent gene and protein pairs. **F** KEGG annotation of expression-consistent gene and protein pairs. GART, GAR formyl transferase. ADK, adenosine kinase. SAICARS, SAICAR synthetase. SAMS, SAM synthetase. PPAT, PRPP amidotransferase. GSDA, guanosine deaminase. XDH, xanthine dehydrogenase.  NMT, *N*-methyltransferase. AMPD, AMP deaminase. ALNS, allantoin synthase. UAH, ureidoglycolate amidohydrolase. APRT, adenine phosphoribosyltransferase. MS, methionine synthase.

To understand the regulatory mechanisms of structural genes in the purine alkaloid metabolic pathway of tea plant under strong light signal, the transcript and protein levels of LT1 and LT4 samples were quantified by integrating transcriptome and proteome sequencing. A total of 6281 expressed gene and protein pairs (EGPPs) were detected from the transcriptome and proteome. By comparing the quantitative data of gene transcription and protein expression, it was found that the fluctuation of protein expression induced by strong light signal was smaller than that of gene transcription (Fig. 2A). Overall, there was a highly significant positive correlation between gene transcription and protein expression (*P* < 0.01), and 60.41% of EGPPs were consistently expressed (Fig. 2B). By screening of thresholds, 7290 differentially expressed genes (DEGs) and 719 differentially expressed proteins (DEPs) were obtained from the transcriptome and proteome, respectively (Fig. 2C). The number of up- and down-regulated DEGs was almost equal in transcriptome, while significantly more DEPs were down-regulated than up-regulated in proteome.

The EGPPs were further classified in a nine-quadrant graph based on expression thresholds (Fig. 2D). The data showed a linear distribution in general. The most data were distributed in quadrant 5 (52.25%), followed by quadrant 2 (15.78%) and quadrant 8 (7.90%). The least data were distributed in quadrant 1 (1.45%) and quadrant 9 (0.51%). The data for EGPPs expression concordance were located in quadrant 3 (5.62%) and quadrant 7 (4.36%). A total of 39 EGPPs were detected in the purine alkaloid metabolism related pathway of tea plant, of which 25 EGPPs were located in quadrant 5 and 14 EGPPs in other quadrants of the nine-quadrant diagram. Only guanosine deaminase (GSDA), XDH, and *S*-adenosyl-L-methionine synthetase1 (SAMS1) were found to be located in quadrant 3. Gene ontology (GO) (Fig. 2E) and kyoto encyclopedia of genes and genomes (KEGG) (Fig. 2F) annotations were performed for EGPPs with consistent expression in the nine-quadrant graph (quadrant 3 and 7). In GO annotations (Fig. 2E), the top 25 GO terms based on *P*-value involved ‘Cellular Component’ and ‘Biological Process’. The GO terms of ‘Cellular Component’ were mainly localized in chloroplast (5) and plastid (5), and secondarily to cytoplasm (2). The GO terms of ‘Biological Process’ involved ‘response to karrikin’, ‘amino acid catabolic process’, ‘pigment metabolic/biosynthetic process’, ‘response to abiotic stimulus/stress’, ‘cofactor metabolic process’, ‘oxidation–reduction process’, and ‘single-organism metabolic/biosynthetic process’. GO terms for ‘Molecular Function’ involved ‘oxidoreductase activity’ and ‘alpha-galactosidase activity’. In KEGG annotation (Fig. 2F), the top 25 KEGG pathways based on *P*-values all belong to the category of ‘Metabolism’. The top two were ‘metabolic pathways’ and ‘biosynthesis of secondary metabolites’, which were the most annotated pathways and belong to ‘the global and overview maps’. We noticed that the subsequent pathways, including ‘Flavonoid biosynthesis’, ‘Phenylpropanoid biosynthesis’, and ‘Flavone and flavonol biosynthesis’, belong to ‘biosynthesis of secondary metabolites’. KEGG annotation also involved photosynthesis, porphyrin metabolism, and several primary metabolic processes.

### Screening and analysis of differential metabolites using metabolomic assay

**Figure 3:**
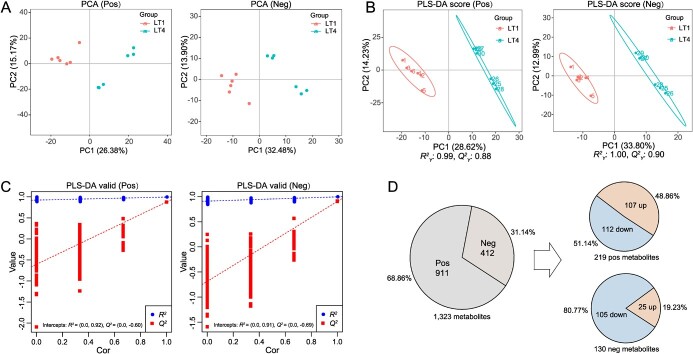
Metabolome analysis in light-treated tea plants. **A** PCA analysis for treatment group (LT4) versus control group (LT1). **B** PLS-DA score for LT4 versus LT1. **C** PLS-DA permutation test for LT4 versus LT1. **D** Screening for differential metabolites. Pos, positive ionization mode. Neg, negative ionization mode.

To investigate the correlation between light intensity and the changes of metabolite components, metabolites in treatment (LT4) and control (LT1) groups were assayed by a non-targeted metabolomic approach. Ultimately, all assay signals were matched to a total of 1323 metabolites. The obtained metabolites were tested separately using multivariate statistical methods, including principal component analysis (PCA) analysis (Fig. 3A), partial least squares discriminant analysis (PLS-DA) score calculation (Fig. 3B), and PLS-DA permutation test (Fig. 3C), respectively. The PCA results showed that the cumulative contribution rates of PC1 and PC2 in positive and negative ionization mode were 41.55% and 46.38%, respectively (Fig. 3A). The within-group data were more concentrated in the control (LT1) and treatment (LT4) groups, indicating that light intensity influenced the changes in the metabolite fractions of tea plants. To verify the accuracy of the model, the samples were re-analysed using supervised PLS-DA [36]. All samples were within the 95% confidence interval, and the samples between LT4 and LT1 groups could be clearly distinguished. The model evaluation parameters (*R*^2^, *Q*^2^) obtained after 7-fold cross-validation, and if *R*^2^ and *Q*^2^ are closer to 1, the model is more stable and reliable [37]. The results showed that *R*^2^ = 0.99 and *Q*^2^ = 0.88 in the positive ionization mode and *R*^2^ = 1.00 and *Q*^2^ = 0.90 in the negative ionization mode (Fig. 3B). This indicated that the PLS-DA model could well explain and predict the differences in metabolite changes in tea plants caused by light intensity treatments. In addition, the model was subjected to ranking validation to test whether the model was over-fitted [37]. Regression lines were plotted through the *Q*^2^ and *R*^2^ values after 200 permutations and modeling. The Y-axis intercepts of *R*^2^ were greater than 0.9 in both positive and negative ionization modes, and the Y-axis intercepts of *Q*^2^ were less than 0, indicating that the model was not over-fitted and the statistical conditions were satisfied for further differential metabolite screening (Fig. 3C). Differential metabolites were screened by referencing parameters, including variable importance in projection (VIP), fold change (FC), and *P*-value, and thresholds of ‘VIP > 1.0, FC > 1.2’ or ‘FC < 0.833, *P*-value < 0.05’. This resulted in 219 and 130 differential metabolites being screened from the positive and negative ionization modes, respectively (Fig. 3D; Table S2, see online supplementary material).

**Figure 4:**
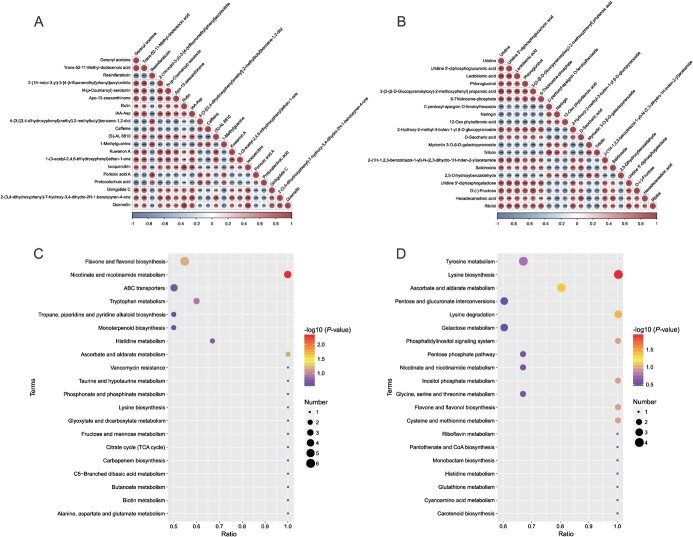
Analysis of differential metabolite association and KEGG annotation. **A** Top 20 differential metabolite association analysis for positive ionization mode (based on *P*-values). **B** Top 20 differential metabolite association analysis for negative ionization mode (based on *P*-values). **C** Top 20 KEGG pathway matched with differential metabolite in positive ionization mode. **D** Top 20 KEGG pathway matched with differential metabolite in negative ionization mode.

The correlations of top 20 differential metabolites (based on *P*-values) were calculated to provide a preliminary understanding of the types of different metabolites and the synergistic or mutually exclusive relationships between individuals (Fig. 4A and B). Many of the differential metabolites were flavonoids, mostly flavonols, such as rutin, quercetin, isoquercitrin, and myricetin-3-O-*β*-D-galactopyranoside, and all were up-regulated as if by a common mechanism. In addition to flavonoids, a variety of metabolites related to tea sensory quality (color, aroma, and taste) were regulated, such as the yellow pigment apo-13-zeaxanthinone, geranyl acetone with floral, and woody aroma were up-regulated, and sweet fructose and bitter caffeine were down-regulated. Differential metabolites were also involved in hormonal regulation. Two signaling molecules, IAA-Asp and 12-oxo phytodienoic acid, associated with auixn and jasmonic acid were up-regulated.

The differential metabolites were known to be involved in 55 metabolic pathways (Tables S3 and S4, see online supplementary material). Based on *P*-values for the matching degree of differential metabolites to metabolic pathways, we ranked these pathways and listed the top 20 pathways (Fig. 4C and D). Flavonoid and flavonol biosynthetic pathways account for the most annotated metabolites, including rutin, quercetin, myricetin, laricitrin, kaempferol, syringetin, trifolin, and luteolin. Except for laricitrin, the other seven metabolites were up-regulated in strong light. Differential metabolites also involved other metabolic pathways, such as ascorbate and aldarate metabolism, nicotinate and nicotinamide metabolism, lysine biosynthesis, ABC transporters, and tyrosine metabolism, all of which were matched to more than three metabolites. Interestingly, at least half of the metabolites were down-regulated in each of these metabolic pathways.

### Metabolic network of the purine alkaloids of tea plant in strong light signal

**Figure 5:**
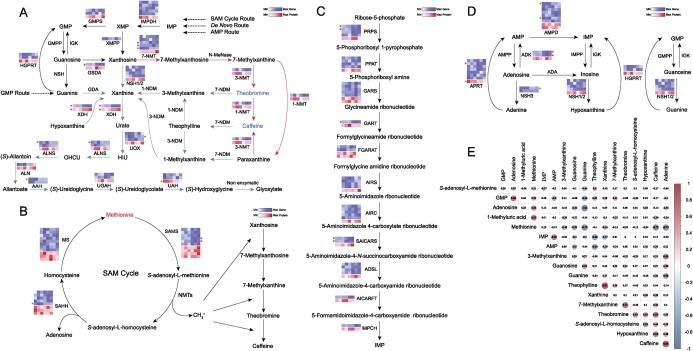
Pathways related to the metabolism of purine alkaloids in tea plant. **A** Caffeine metabolic pathway. **B** SAM pathway. **C***de novo* pathway. **D** Salvage pathway. **E** Clustering heat map of in pathways related to the metabolism of purine alkaloids. Red and blue type represent up-regulated and down-regulated differential metabolites, respectively. In the expression color blocks, the first three are from LT1, and the last three are from LT4. Red and blue stars represent up- and down-regulation of DEGs or DEPs, respectively. Double arrows represent omitted pathways, while dashed lines represent the source or destination of metabolites. Red arrows represent the biosynthetic pathway of caffeine, while blue arrows represent the degradation pathway of caffeine. GMPP, GMP phosphatase. IGK, inosine/guanosine kinase. NSH, nucleoside hydrolase. HGPRT, hypoxanthine/guanine phosphoribosyltransferase. GMPS, GMP synthase. XMPP, XMP phosphatase. GSDA, guanosine deaminase. NSH, nucleoside hydrolase. IMPDH, IMP dehydrogenase. 7-NMT, *N7*-methyltransferase. N-MeNase, *N*-methylnucleotidase. 3-NMT, *N3*-methyltransferase. 1-NMT, *N1*-methyltransferase. 7-NDM, methylxanthine *N7*-demethylase. 3-NDM, methylxanthine *N3*-demethylase. 1-NDM, methylxanthine *N1*-demethylase. GDA, guanine deaminase. XDH, xanthine dehydrogenase. UOX, urate oxidase. ALNS, allantoin synthase. ALN, allantoinase. AAH, allantoate amidohydrolase. UGAH, ureidoglycine aminohydrolase. UAH, ureidoglycolate amidohydrolase. SAHH, SAH hydrolase. MS, methionine synthase. SAMS, SAM synthetase. NMTs, *N*-methyltransferases. PRPS, PRPP synthetase. PPAT, PRPP amidotransferase. GARS, GAR synthetase. GART, GAR formyl transferase. FGARAT, FGAR amidotransferase. AIRS, AIR synthetase. AIRC, AIR carboxylase. SAICARS, SAICAR synthetase. ADSL, adenylosuccinate lyase. AICARFT, AICAR formyltransferase. IMPCH, IMP cyclohydrolase. AMPP, AMP phosphatase. ADK, adenosine kinase. APRT, adenine phosphoribosyltransferase. AMPD, AMP deaminase. ADA, adenosine deaminase. IMPP, IMP phosphatase.

To investigate the effect of light intensity on the regulation of purine alkaloid metabolic network in tea plant, multi-omics data were integrated to analyse the pathways including caffeine metabolism (Fig. 5A), *S*-adenosyl-L-methionine (SAM) cycle (Fig. 5B), *de novo* purine biosynthesis (Fig. 5C), and purine salvage (Fig. 5D). Three differential metabolites (Table S5, see online supplementary material), 22 DEGs (Table S6, see online supplementary material), and three DEPs (Table S7, see online supplementary material) were detected in these pathways. Metabolomic data showed that caffeine and theobromine were significantly reduced and methionine was significantly increased by strong light.

**Figure 6:**
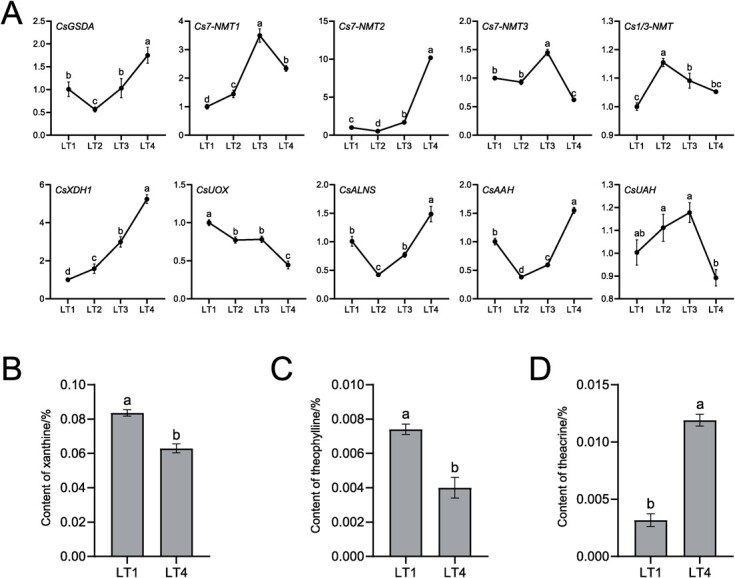
Detection of key genes and metabolites in the metabolic pathway of purine alkaloids. **A** Expression of key genes. **B** Xanthine content in LT1 and LT4 treatment. **C** Theophylline content in LT1 and LT4 treatment. **D** Theacrine content in LT1 and LT4 treatment. GSDA, guanosine deaminase. 7-NMT, *N7*-methyltransferase. 1/3-NMT, *N1/3*-methyltransferase. XDH, xanthine dehydrogenase. UOX, urate oxidase. ALNS, allantoin synthase. AAH, allantoate amidohydrolase. UAH, ureidoglycolate amidohydrolase.

Starting from xanthosine, a total of seven DEGs and two DEPs were detected in the biosynthesis and catabolism of caffeine (Fig. 5A). Four DEGs, including three up-regulated and one down-regulated, were involved in caffeine biosynthesis. In caffeine catabolism, XDH is the only one that is significantly up-regulated at both gene and protein levels. In contrast, the catalytic enzymes differentially expressed in urate catabolic pathway were down-regulated at either the gene or protein level. Based on the possibility that DEGs, DEPs, and caffeine synthase genes play important roles in caffeine metabolism, the expression of DEGs, DEPs corresponding genes, and *Cs1/3-NMT* gene were further verified by qRT-PCR. The results showed that the expression trends of these genes were consistent between omics and qRT-PCR analyses, except for *CsALNS* (encoding allantoin synthase) and *CsAAH* (encoding allantoate amidohydrolase) genes (Fig. 6A). Remarkably, the expression of *CsXDH1* gene was up-regulated with increasing light intensity, which was negatively correlated with the trend of caffeine and theobromine in response to light intensity.

In the methyl-derived SAM cycle, two up-regulated DEGs encoding *S*-adenosyl-L-methionine synthetase (SAMS) and one down-regulated DEG encoding *S*-adenosyl-L-homocysteine hydrolase (SAHH) were detected (Fig. 5B). The trend of SAMS protein expression was up-regulated, while SAHH protein expression tended to be down-regulated. This suggests that SAMS may enhance the conversion of methionine to *S*-adenosyl-L-methionine in SAM cycle, thereby facilitating the supply of methyl donors. In addition, four DEGs were detected in *de novo* purine biosynthesis, and four DEGs and one DEP were detected in purine salvage (Fig. 5C and D). From the expression data, it appears that *de novo* purine biosynthesis may be enhanced in continuous strong light, while the regulation trend of purine salvage was not obvious.

The correlation of detected 18 metabolites was analysed to understand the synergistic or mutually exclusive relationships between different metabolite individuals (Fig. 5E). The metabolites positively correlated (*r* > 0.3) with caffeine were adenine, theobromine, hypoxanthine, *S*-adenosyl-L-homocysteine, xanthine, guanine, AMP, and 3-methylxanthine, while the negatively correlated (*r* < −0.3) metabolites were methionine and 1-methyluric acid. The metabolites positively correlated (*r* > 0.3) with theobromine were 7-methylxanthine, caffeine, *S*-adenosyl-L-homocysteine, hypoxanthine, adenine, and guanosine, while the negatively correlated metabolites were 3-methylxanthine (*r* = −0.25) and methionine (*r* = −0.22). These results suggest that the metabolites in caffeine, theobromine, and the metabolites in their adjacent metabolic pathway branches are related to each other. As important metabolites for the continued demethylation or methylation of caffeine, xanthine, theophylline, and theacrine were further examined by HPLC (Fig. S1, see online supplementary material). The results showed that theophylline and xanthine decreased significantly in strong light, and theacrine increased significantly under the same conditions (Fig. 6B, C, and D). The increase in theacrine may be related to the up-regulated expression of the genes encoding *N*-methyltransferases, while enhanced caffeine catabolism leads to a decrease in theophylline and xanthine.

### *CsXDH1* gene overexpression induced by strong light promotes the degradation of caffeine, theobromine, and xanthine in tea plant

**Figure 7:**
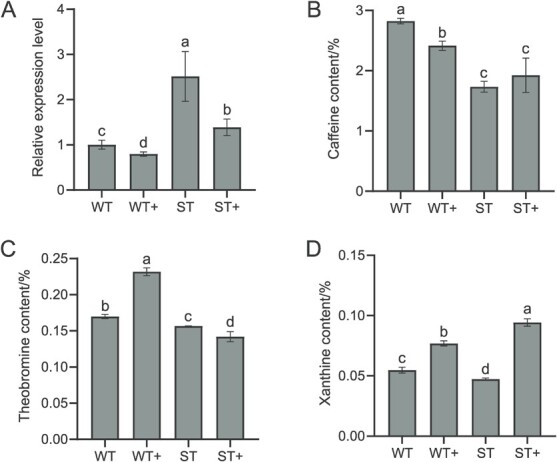
asODN silencing test. **A** Effect of asODN silencing on *CsXDH1* gene expression. **B** Effect of silencing *CsXDH1* gene on caffeine. **C** Effect of silencing *CsXDH1* gene on theobromine. **D** Effect of silencing *CsXDH1* gene on xanthine. WT, samples under weak light. WT+, samples of silencing *CsXDH1* gene under weak light. ST, samples under strong light. ST+, samples of silencing *CsXDH1* gene under strong light.

XDH induced by strong light may play a key role in catalyzing the degradation of purine alkaloids of tea plant. To verify this inference, this study employed an antisense oligodeoxynucleotide chain (asODN) gene-specific interference technique to silence the expression of gene *CsXDH1*. After asODN silencing, the relative expression level of *CsXDH1* gene in weak light treatment (WT) and strong light treatment (ST) were significantly reduced by 20.58% and 44.75%, respectively (Fig. 7A). *CsXDH1* gene expression were consistently significantly higher in ST samples than in WT samples, regardless of silencing or non-silencing treatments. It showed that *CsXDH1* gene expression was suppressed by asODN silencing and the effect was more pronounced in ST samples.

The contents of caffeine, theobromine, and xanthine in test samples were examined to investigate how asODN silencing affects the degradation process of caffeine in tea plant. The results showed that asODN silencing significantly reduced the caffeine content of WT samples by 14.51%, while it did not reduce the caffeine content of ST samples, and even increased the content by 11.06% (Fig. 7B). The effect of asODN silencing on theobromine content followed the opposite trend to that of caffeine. After asODN silencing, the content of theobromine in WT samples increased significantly by 36.51%, while for ST samples the content of theobromine decreased by 9.38% (Fig. 7C). In contrast, the effect of asODN silencing on xanthine was more prominent. Under the same conditions, the content of xanthine in WT and ST samples increased significantly by 40.51% and 98.95%, respectively (Fig. 7D).

**Figure 8:**
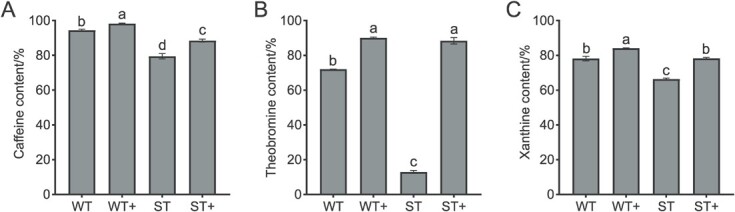
*In vitro* enzymatic reaction. **A** Effect of silencing *CsXDH1* gene on caffeine degrading enzyme activity. **B** Effect of silencing *CsXDH1* gene on theobromine degrading enzyme activity. **C** Effect of silencing *CsXDH1* gene on xanthine degrading enzyme activity. WT, samples under weak light. WT+, samples of silencing *CsXDH1* gene under weak light. ST, samples under strong light. ST+, samples of silencing *CsXDH1* gene under strong light.

**Figure 9:**
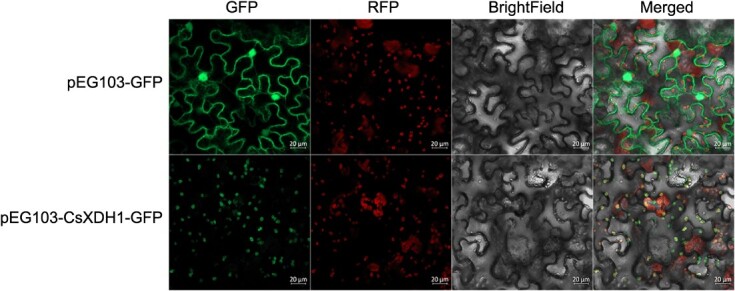
Subcellular localization of CsXDH1 in tobacco cells.

The effect of asODN silencing was further verified by *in vitro* assay in order to avoid possible interference with cellular factors. The results showed that the enzymatic activity efficiency of ST sample was higher than that in WT sample in non-silencing treatments, while asODN silencing attenuated the degradation of exogenous caffeine, theobromine, and xanthine by the protease (Fig. 8A, B, and C). In these groups of tests, asODN silencing reduced the enzyme activity in the samples by 27.36% to 86.64%. Therefore, the enzymatic activity of CsXDH1 may be influenced by cell factors. The subcellular localization of CsXDH1 was further observed by constructing a pEG103-CsXDH1-GFP fusion expression plasmid, and by transferring into tobacco cells. Fluorescence signals showed that the pEG103-CsXDH1-GFP co-localized precisely with auto-fluorescence signals in chloroplasts (Fig. 9). This suggests that CsXDH1 is a chloroplast localization protein.

## Discussion

### Co-analysis of multi-omics yielded more comprehensive information

Tea plant is a photosensitive plant and its agroecological concept of light management has been known and applied for more than 1000 years [30]. It is only in recent years that some mechanisms for the operation of light regulation in tea plant have been found through cytology, biochemistry, and molecular biology [30, 32, 33, 38, 39]. Single omics has been used in almost all studies of light intensity regulation in tea plants, which provides a great deal of information for mechanism elucidation [32, 33, 40]. However, single omics has limitations in the comprehensiveness and objectivity of information [41]. For example, the transcriptome does not fully reflect the true picture of proteins because the mRNA transcription to protein translation process is regulated by variable shear, post-translational modification, and degradation of proteins, while proteomics assays are less sensitive and particularly difficult to detect low abundance proteins [41]. Therefore, co-analysis of multi-omics is believed to provide more comprehensive information in biomolecular regulation studies [41]. This study combined transcriptome, proteome, and metabolome to focus on the light adaptation, tea quality compounds, and caffeine metabolism of tea plant in variable light intensities. The following points are not difficult to find in the overall assessment of the multi-omics data. mRNA transcript levels fluctuated more than protein levels, and a large number of uncorrelated, negatively, and positively correlated EGPPs were observed. Overall EGPPs were positively correlated (*r* = 0.43, *P* < 0.01) reflecting that there is indeed an intrinsic link between transcript levels and protein levels, which is consistent with previous studies in many plants [42–44]. EGPPs and metabolome annotations are associated, and the regulation of metabolites can be supported from EGPPs. These indicate that the integration of multi-omics is necessary for the mutual support and accurate interpretation of the data.

### Adaptation strategies of tea plant response to light intensity

The difference factors obtained from the multi-omics analysis of this study reflected the adaptive strategies of tea plant to light intensity. Photosynthetic organs in plants are the most sensitive organelles to light intensity and can adapt to light intensity changes through adjustments in chloroplast numbers, photosynthetic system structure, and components [45]. The results in the multi-omics annotation based on threshold analysis showed that chloroplasts and plastids were the most localized organelles for GO terms, and the photosynthesis and porphyrin metabolism were the prominent response pathways in the KEGG annotation, which support the previous conclusions. Strong light stress is prone to induce the accumulation of reactive oxygen species (ROS) in plants, which will lead to oxidative damage to cells and affect protein function [46, 47]. In this study, several coping strategies for increased ROS caused by strong light were detected in tea plants. Flavonols are effective ROS scavengers and filters of harmful light radiation [48]. Multi-omics results showed that flavonoids, especially flavonols, are a prominent type of compounds regulated by differential light intensities, suggesting that flavonols might play important roles in avoiding strong light damage. Defense enzymes were actively involved in the metabolism to prevent excessive accumulation of ROS in strong light (Fig. S2, see online supplementary material). In addition, we observed that ascorbic acid (Asc) and glutathione (GSH) as low molecular weight antioxidants were up-regulated. Asc is a direct antioxidant and also acts as a substrate for ascorbate peroxidase (APX) to detoxify H_2_O_2_ into water [45, 49]. GSH is oxidized by ROS in oxidative stress as part of the antioxidant barrier that weakens excessive oxidation of sensitive cellular components [50].

### Caffeine catabolism in tea plant was enhanced in continuous strong light

In a previous ^14^C tracing experiment [51], caffeine catabolism in tea leaves was susceptible to light environment relative to shading. However, the catabolism of tea caffeine induced by light environment is performed slowly, and the apparent differences can be observed only after a long-term amount of superposition [51]. The conversion of caffeine to theophylline is thought to be the rate-limiting step that triggers caffeine accumulation [19]. Another reason why caffeine is difficult to be degraded in tea may be related to the metabolic circuit of caffeine catabolism. Based on feeding experiments [19, 22], several potential metabolic circuits exist among, but not limited to, caffeine, theobromine, theophylline, and 3-methylxanthine, which may avoid the needless entry of these alkaloids into xanthine catabolism. This study found that caffeine, theobromine, and the metabolites in their adjacent metabolic pathway branches, including 3-methylxanthine, 7-methylxanthine, xanthine, and hypoxanthine, tended to be down-regulated in strong light based on long-term observations (14 d). The possibility is that the decrease in purine alkaloids caused by strong light is attributed to inhibited biosynthesis or enhanced catabolism. Multi-omics showed that the *de novo* pathway and methyl supply of purine alkaloids may be enhanced in strong light. Moreover, strong light induced up-regulation of the expression profile of several genes encoding *N*-methyltransferases of caffeine biosynthesis in qRT-PCR tests. These results did not support well that strong light inhibited caffeine biosynthesis. Therefore, it is speculated that purine nucleotides in continuous strong light tend to be used for nucleic acid and energy metabolism via catabolism rather than biosynthesis, as suggested by Suzuki [51].

### CsXDH1 may be a coordinating factor between light intensity adaptation and caffeine catabolism

XDH (EC: 1.17.1.4) is a molybdenum-containing flavoenzyme that catalyzes the successive oxidation of hypoxanthine to xanthine and xanthine to urate in purine catabolism. It has been found that XDH-catalyzed intermediates in plant were involved in stress regulation through ROS metabolism and nitrogen transport [52–54]. The strict correspondence between *CsXDH1* expression and gradient light intensity suggests that tea plant may enhance purine metabolism through XDH to adapt to strong light stress. This was supported by asODN silencing and *in vitro* enzyme activity assays for xanthine. Interestingly, the effect of *CsXDH1* silencing on caffeine and theobromine was not as pronounced in *in vivo* assay as it was in *in vitro* assay. Only theobromine was significantly up-regulated in the silencing treatment of WT whether in *in vivo* or *in vitro* assay. It indicates that caffeine catabolism in strong light is slow, probably due to limitation by certain factors in cells rather than enzymatic activity. Not coincidentally, a previous short-term *in vivo* test using allopurinol to silence XDH found that silencing caused up-regulation of theobromine and reduced the conversions of theobromine to caffeine [19], which is consistent with the WT results of this study. XDH1 is a membrane associated protein in *Arabidopsis thaliana* and localized to the tonoplast, and its localization is closely associated with chloroplasts [53]. The slight difference was found that CsXDH1 fusion protein was localized to chloroplast in tobacco. The subcellular localization of caffeine in tea plant has not been conclusively demonstrated and is thought to be possibly located in the vesicles of leaf tissue and associated with chloroplast localization [1]. It is speculated that CsXDH1 may act as an important coordinator in light intensity adaptation to the extent that the process of caffeine catabolism is modified, which would otherwise be difficult in tea plant.

## Materials and methods

### Plant materials, growth conditions, and light treatments

The one-year-old seedlings of tea cultivar ‘Fuding Dabaicha’ were purchased from Miaoyi Agriculture Co., Ltd, Yichang, China. The growth soil of tea seedlings was composed of peat moss and vermiculite in a volume ratio of 3:1. The temperature, humidity, and light conditions for growth were as follows: the ambient temperature was maintained at 23 ± 1°C; the humidity was about 70%; using visible full-spectrum LED light sources to simulate 16 h daytime and 8 h nighttime in a day. All tea seedlings were pretreated for one month under light intensity with 160 μmol·m^−2^·s^−1^. Subsequently, healthy tea seedlings with no significant difference were selected as treatment materials and divided into four groups. More than 500 seedlings were used for each group of treatments. The four groups of materials were illuminated with light intensity of 15 μmol·m^−2^·s^−1^ (LT1), 160 μmol·m^−2^·s^−1^ (LT2), 200 μmol·m^−2^·s^−1^ (LT3), and 240 μmol·m^−2^·s^−1^ (LT4), respectively. The light intensity settings were based mainly on the detection of chlorophyll fluorescence parameters (Table S8, see online supplementary material) and references [55–57]. Other conditions of these groups were the same as pretreatment. After two weeks, the shoots, including one bud and two leaves, of tea plants were harvested for testing. All samples except those specifically noted were observed and tested by performing three biological and three technical replicates.

### Chlorophyll detection

An amount of 0.1 g (accurate to 0.0001 g) of each tea sample was weighed and added to 1 mL of pure water for grinding. The entire grinding solution was further prepared by anhydrous ethanol and acetone in a volume ratio of 1:2 to 10 mL. The mixture prepared above was treated in the dark for 3 h until the tissue was nearly white and then used for chlorophyll detection. The absorbance values of chlorophyll at 645 nm and 663 nm were detected by ReadMax 1200 microplate reader. Chlorophyll *a* content (mg/g) = (12.7 × A_663_−2.69 × A_645_) × V × F ÷ W ÷ 1000. Chlorophyll *b* content (mg/g) = (22.9 × A_645_−4.68 × A_663_) × V × F ÷ W ÷ 1000. Total chlorophyll content (mg/g) = (20.21 × A_645_ + 8.02 × A_663_) × V × F ÷ W ÷ 1000. V: volume of extraction solution, 10 mL. F: dilution multiple. W: sample mass, g.

### Determination of purine alkaloids by HPLC

The tea samples were extracted by the extraction method [58]. The steps were as follows: microwave fixed for 2 min, dried in an oven at 60°C until dry enough, and sieved with a 30-mesh sieve after grinding; 0.2 g (accurate to 0.0001 g) of each sieved sample was weighed and put into ultrapure water (5 mL) preheated at 70°C, and then fully wetted and shaken; subsequently, the samples were transferred to a 70°C water bath for 10 min (stirring every 5 min), repeated once (4 mL ultrapure water was added for the second time), and then cooled at room temperature; each extract was centrifuged at 3500 r/min (10 min) to obtain the supernatant, and then the volume was made up to 10 mL with ultrapure water for quantitative analysis of caffeine, theobromine, theophylline, theacrine, and xanthine.

The sample extract was filtered through 0.22 μm of membrane and sampled onto 1220 Infinity II LC for detection. Purine alkaloid standards were purchased from Feiyu Biotechnology Co., Ltd, Jiangsu, China and Amida Biotechnology Co., Ltd, Chongqing, China. Acetonitrile and glacial acetic acid were both HPLC grade, filtered through 0.22 μm membrane before use. The conditions were as follows: column was Agilent ZORBAX SB-C18; 274 nm UV wavelength; mobile phase A was 0.2% glacial acetic acid; mobile phase B was pure acetonitrile; 0.9 mL/min flow rate; temperature at 25°C; 20 μL per injection. The gradient elution procedure was as follows: 8 ~ 16% B, 0 ~ 10 min; 16 ~ 20% B, 10 ~ 20 min; 20 ~ 8% B, 20 ~ 25 min; 8% B, 25 ~ 30 min.

Caffeine, theobromine, theophylline, theacrine, and xanthine standards were accurately weighed, and then dissolved in pure acetonitrile for preparing the standard stock solution (1 mg/mL). After the stock solution was diluted 25 times with ultrapure water, the single standard of purine alkaloids was obtained; the standard stock solutions of purine alkaloids were mixed with equal volume and then diluted 25 times with ultrapure water to prepare a mixed standard solution.

### Transcriptomic analysis

RNA was extracted from samples (LT1 and LT4) using Quick RNA Isolation Kit. RNA integrity and purity of samples were analyzed by NanoPhotometer and Agilent 2100. NEBNext^®^ Ultra™ RNA Library Prep Kit for Illumina^®^ was used to construct the library, and sequence data (reads) were obtained by Illumina sequencing. Clean reads were mapped using HISAT2 to generate the positioning information of reads on the reference genome. StringTie was used for assembling the mapped reads of each sample. The number of reads mapped to each gene was analysed by featureCounts. Expression difference DESeq2 software was used to calculate differential gene expression. FDR ≤ 0.05 and |log_2_(FC)| > 1.5 were the threshold values for screening DEGs.

### Proteomics analysis

Protein was extracted from samples (LT1 and LT4) using the cold acetone method. Bovine serum albumin (BSA) method was used to measure protein concentration. The protein samples were separated using 12% SDS-PAGE gel electrophoresis. Proteins after quantification were digested with trypsin, and passed through desalting column, and then labeled with TMT reagent. The labeled samples were mixed by equal volume, and then desalted and lyophilized. 2% acetonitrile was used to dissolve the lyophilized samples.

L3000 HPLC system with Waters BEH C18 column was used to separate fractions. The conditions were as follows: Monitoring of eluates at UV 214 nm; mobile phase A was 2% acetonitrile (pH 10.0); mobile phase B was 98% acetonitrile; 1 mL/min flow rate; temperature at 50°C. The elution procedure was as follows: 3 ~ 5% B, 0 ~ 10 min; 5 ~ 20% B, 10 ~ 30 min; 20 ~ 40% B, 30 ~ 48 min; 40 ~ 50% B, 48 ~ 50 min; 50 ~ 70% B, 50 ~ 53 min; 70 ~ 100% B, 53 ~ 54 min. The fractions were lyophilized and dissolved with 0.1% formic acid.

Mobile phase A (100% water, 0.1% formic acid) and mobile phase B (80% acetonitrile, 0.1% formic acid) were prepared. EASY-nLC™ 1200 UHPLC system with home-made C18 nano-trap column and home-made analytical column was used to liquid chromatography detection. The elution procedure was as follows: 6 ~ 17% B, 0 ~ 2 min; 17 ~ 40% B, 2 ~ 52 min; 40 ~ 55% B, 52 ~ 54 min; 55 ~ 100% B, 54 ~ 55 min; 100% B, 55 ~ 60 min.

Q Exactive HF-X were used to mass spectrometry detection. The ion source was from Nanospray Flex™ (ESI) with 2.3 kV spray voltage and 320°C ion transport capillary temperature. Full scan range, primary mass spectrometry resolution, automatic gain control (AGC) target value, and maximum ion injection time were set to *m*/*z* 350–1500, 6 × 10^4^ (at *m/z* 200), 3 × 10^6^, and 20 ms, respectively. The top 40 highest abundant precursors of full scan were fragmented by higher energy collisional dissociation (HCD) for the secondary mass spectrometry where resolution was 3 × 10^4^ (*m/z* 200) for 6 plex. AGC target value, dynamic exclusion parameter, maximum injection ion time, normalized collision energy, intensity threshold, and were set to 5× 10^4^, 20 s, 54 ms, 32%, and 1.2 × 10^5^, respectively.

Raw data were searched separately and filtered using Proteome Discoverer 2.2. The *t*-test was used for statistical analysis. DEPs were selected with |log2(FC)| > 1.2 and *P* < 0.05.

### Metabolomics analysis

Samples of 100 mg which had been ground in liquid nitrogen were vortexed and shaken with aqueous methanol (500 μL of 80%), and then incubated on ice for 5 min. After centrifugation at 15000 *g* (4°C, 20 min), a certain amount of supernatant was diluted with mass spectrometry grade water so that the final methanol concentration was 53%. The supernatant was obtained by centrifugation at 15000 *g* (4°C, 20 min) to prepare the solution to be tested.

Vanquish UHPLC with a Hypesil Gold C18 column was used for liquid chromatographic analysis. The conditions were as follows: mobile phase A were 0.1% formic acid for positive mode and 5 mM ammonium acetate (pH 9.0) for negative mode; mobile phase B was pure methanol; 0.2 mL/min flow rate; temperature at 40°C; 20 μL per injection. The gradient elution procedure was as follows: 2% B, 0 ~ 1.5 min; 2 ~ 100% B, 1.5 ~ 12 min; 100% B, 12 ~ 14 min; 100 ~ 2% B, 14 ~ 14.1 min; 2% B, 14.1 ~ 17 min.

Q Exactive HF-X was used to mass spectrometry analysis. The scan range was set to 100 ~ 1500 *m/z*. Settings of ESI source: 3.2 kV spray voltage; 10 arb aux gas flow rate; 40 arb sheath gas flow rate; 320°C capillary temperature; data-dependent scans as MS/MS secondary scan; polarity: positive/negative.

Compound Discoverer 3.1 was used for peak alignment and peak picking as well as qualitative and quantitative analysis of metabolites by accessing the mzCloud, mzVault, and Masslist database. Metabolites were annotated against the KEGG, HMDB, and LIPIDMaps databas. MetaX software was used for PCA and PLS-DA. Differential metabolites were considered with three parameters, including *P*-value of *t* test, first principal component VIP of PLS-DA, and FC. VIP > 1.0, FC > 1.2 or FC < 0.833, *P*-value <0.05 as thresholds.

### Antisense oligodeoxynucleotide inhibition assay

Antisense oligodeoxynucleotides (asODNs) for *CsXDH1* gene silencing were generated by Primer Premier 5.0. The silent primer sequence used was CsXDH1-asODN (AATGTTTCCCTGGCAATCAA). The shoots of tea seedlings under LT1 and LT4 light conditions were picked and incubated at a solution of 30 μM CsXDH1-asODN primer and 40 μM sucrose in 10 mL centrifuge tubes, subsequently restoring the previous light conditions, and 10 repetitions were set for each group. At the same time, samples were treated in 40 μM sucrose as a control for asODN silencing. To minimize the influence of development degree and uneven absorption of the silencing solution on the results, unfolded one-bud and one-leaf shoots were selected as test samples, and asODN silencing of buds and leaves was separated (Fig. S3, see online supplementary material). After 3 days, asODN silent samples were collected for molecular detection. The asODN silent samples were fixed by microwave fixation and then thoroughly dried in an oven for biochemical assays.

### Gene expression assays

Total RNA was extracted using Quick RNA Isolation Kit. Goldenstar™ RT6 cDNA Synthesis Kit was used for cDNA synthesis. The detection primers of gene expression were designed by Primer Premier 5.0. *CsTIP41* was used as the reference gene for light treatment [59]. qRT-PCR program was performed on the CFX96™ Real-Time System, and the fluorescent reagent was MonAmp™ ChemoHS qPCR Mix. The reaction solution (20 μL) included MonAmp™ ChemoHS qPCR Mix (10 μL), forward primer (0.4 μL, 10 μM), reverse primer (0.4 μL, 10 μM), cDNA (2 μL, 50 ng/μL), and nuclease-free water (7.2 μL). The amplification reaction program was as follows: 95°C for 10 min; 95°C for 10 s, 60°C for 30 s, 40 cycles. The relative expression levels of candidate genes were calculated using the 2^-ΔΔCt^ method [60]. The primer sequences were listed in the Table S9 (see online supplementary material).

### *In vitro* enzymatic reaction

Total proteins of tea samples were extracted by Plant Total Protein Extraction Kit for *in vitro* enzymatic reaction test. The reaction volume for experimental group was 1.1 mL, including 0.5 mL of enzyme solution, 0.3 mL of 0.25 mmol/L substrate solution, and 0.3 mL of ultrapure water. The substrate solution was prepared from caffeine or theobromine or xanthine in 0.02 mol/L, pH 8.0 phosphate buffer. The prepared enzyme reaction solution was reacted at 45°C and pH 8.0 for 30 min, and then trichloroacetic acid (TCA) solution (0.3 mL of 100%) was added to stop. Before the reaction of the experimental group, the TCA solution was added for early termination as control group. After the reaction, the solution was filtered through 0.22 μm membrane filter and immediately stored at −40°C. The content of caffeine, theobromine, and xanthine in the storage solution was detected by HPLC within 3 days, respectively.

### Analysis of subcellular localization

A number of tobacco seeds were sowed and cultured under 12 h of light for one month to be used for this experiment. The constructed vector plasmid (pEG103-CsXDH1-GFP) transformed into agrobacterium EHA105 by electroporation, and cultured at 28°C for 2 d. pEG103-GFP vector was used as a control for test. Individual colonies of agrobacterium were extracted, suspended in YEB liquid medium (10 mL), centrifuged at 200 rpm/min and incubated at 28°C for 2 d. After centrifuging at 5000 rpm/min (4 min), the supernatant was removed to collect the bacterium and then the bacterium was resuspended with 10 mM MgCl_2_ (containing 120 μM acetosyringone) suspension, and adjusted OD_600_ to 0.6. Tobacco were injected from the lower epidermis of tobacco leaves with a 1 mL syringe with the tip removed, and then incubated for 2 d and observed by LSM780.

### Bioinformatics and statistical analysis

The nine-quadrant, GO, and KEGG annotation analyses of EGPPs were performed using the OmicShare tools. The correlation heat map was constructed by Hiplot, the clustering method was average, and the correlation calculation method used spearman. The heat maps of gene and protein expression profiles were drawn through Heatmapper. Statistical analyses were performed by one-way ANOVA using SPSS 21. The treatment means were compared by least significant difference (LSD) with *P <* 0.05.

## Supplementary Material

Web_Material_uhad090

## Data Availability

All relevant data in this study are provided in the article and its supplementary file.
