# Supplementary material for: CsXDH1 gene promotes caffeine catabolism induced by continuous strong light in tea plant
Source: Hortic Res. 2023 May 4;10(6):uhad090. doi: 10.1093/hr/uhad090 (PMC10277909; doi:10.1093/hr/uhad090)
Supplement: Web_Material_uhad090 [file web_material_uhad090.zip › Supplementary Figure S1.pdf]

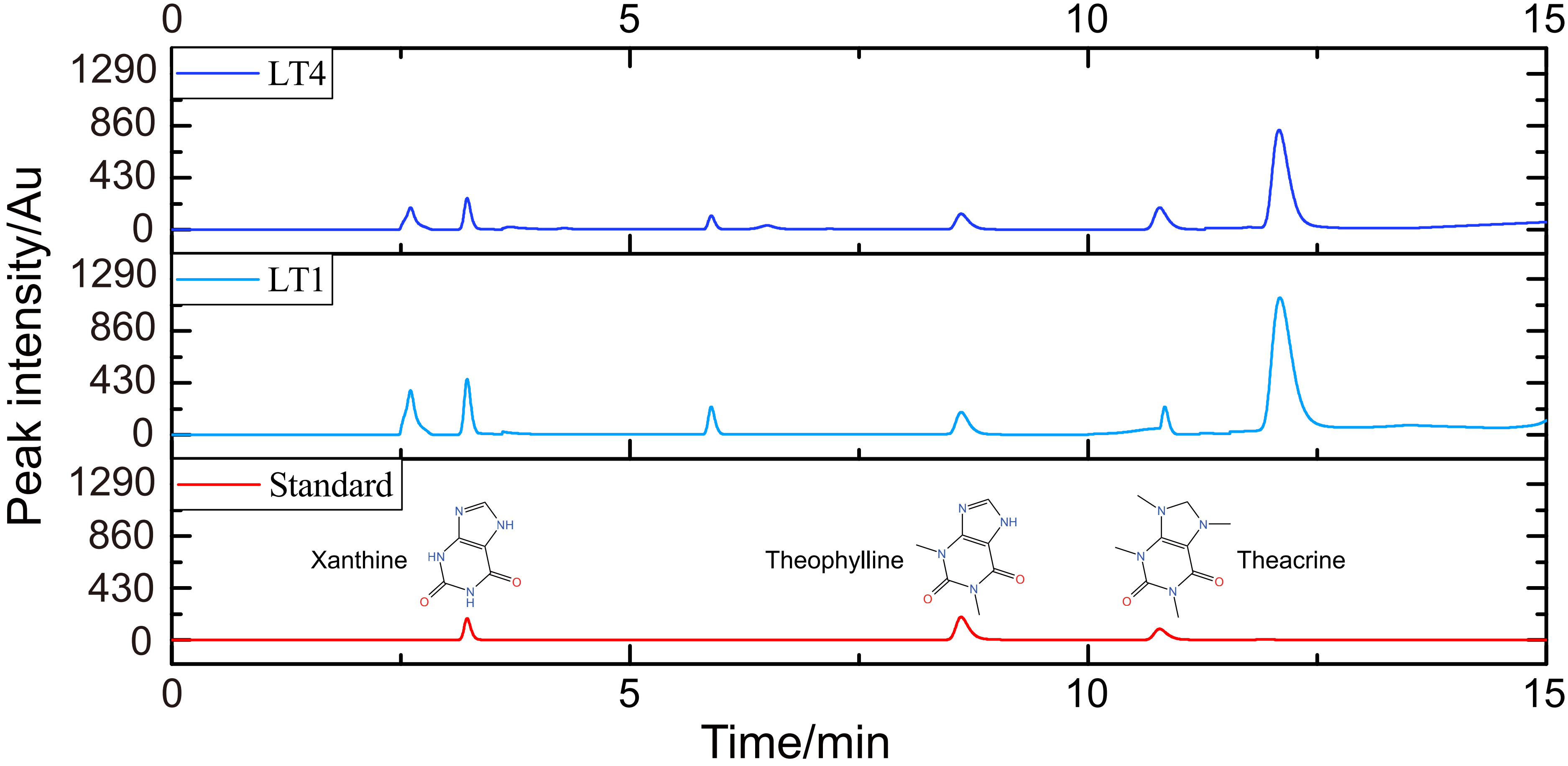

Supplementary Figure S1. Liquid chromatogram of xanthine, theophylline, and theacrine contents in treated samples
