# Supplementary material for: CsXDH1 gene promotes caffeine catabolism induced by continuous strong light in tea plant
Source: Hortic Res. 2023 May 4;10(6):uhad090. doi: 10.1093/hr/uhad090 (PMC10277909; doi:10.1093/hr/uhad090)
Supplement: Web_Material_uhad090 [file web_material_uhad090.zip › Supplementary Figure S2.pdf]

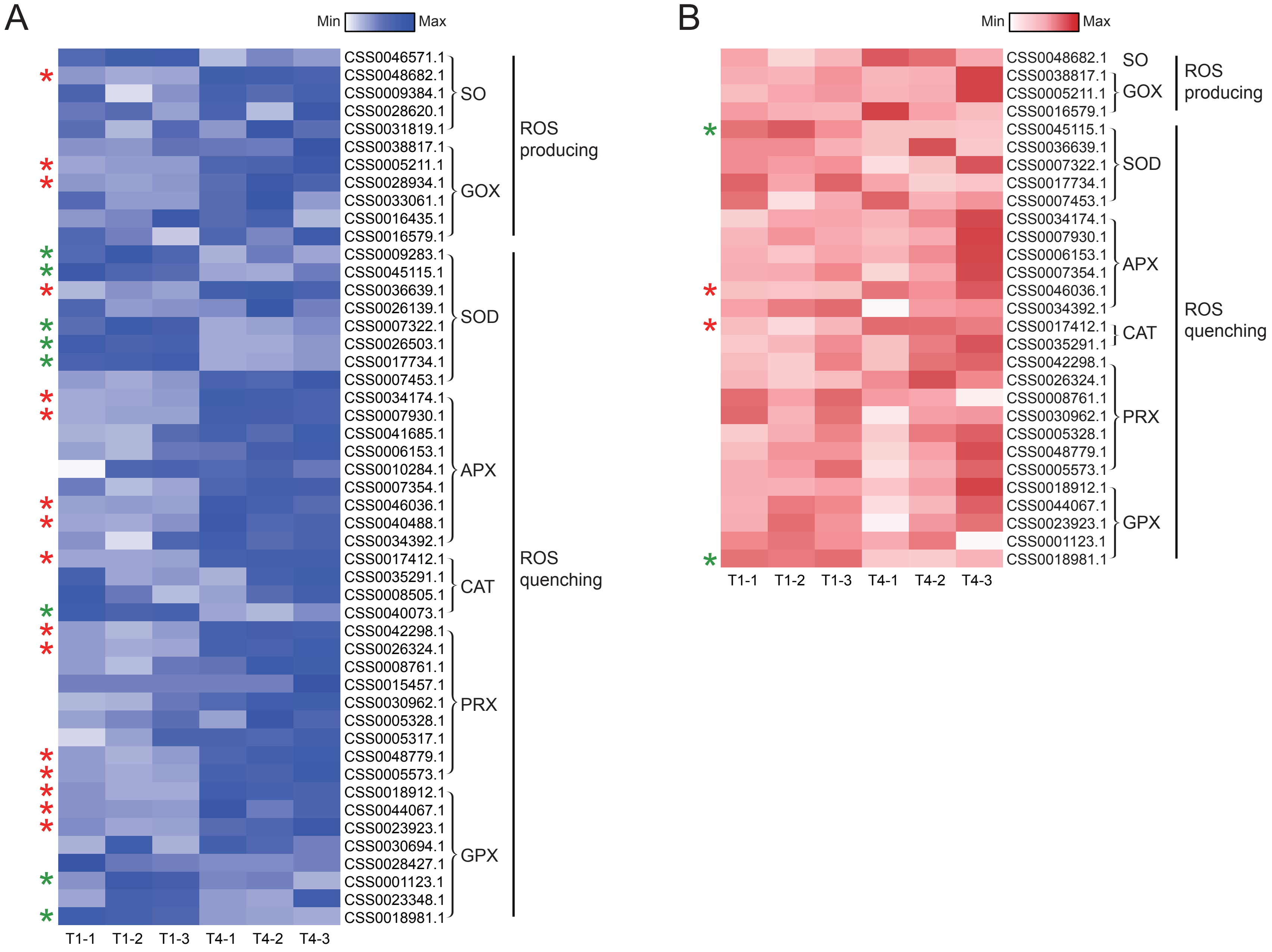

Supplementary Figure S4. Expression profiles of oxidative and antioxidant enzymes in ROS metabolism. **(A)** Gene expression profiles. **(B)** Protein expression profiles. Red and blue stars represent up- and down-regulation of DEGs or DEPs, respectively.
